# Endoplasmic Reticulum Stress: Triggers Microenvironmental Regulation and Drives Tumor Evolution

**DOI:** 10.1002/cam4.70684

**Published:** 2025-03-04

**Authors:** Chaosheng Peng, Juan Wang, Shu Wang, Yan Zhao, Haoyuan Wang, Yuhao Wang, Yuxuan Ma, Jianjun Yang

**Affiliations:** ^1^ Department of Digestive Surgery Xijing Hospital of Digestive Diseases, Fourth Military Medical University Xi'an China; ^2^ State Key Laboratory of Holistic Integrative Management of Gastrointestinal Cancers and National Clinical Research Center for Digestive Diseases Xijing Hospital of Digestive Diseases, Fourth Military Medical University Xi'an China

**Keywords:** endoplasmic reticulum stress, tumor microenvironment, unfolded protein response

## Abstract

**Background:**

The endoplasmic reticulum (ER) serves as a crucial hub for protein synthesis and processing, playing an essential role in maintaining protein homeostasis. Perturbations, such as hypoxia, oxidative stress, inadequate amino acid supply, Ca^2+^ imbalance, and acidosis, can disrupt cellular equilibrium and result in the accumulation of misfolded/unfolded proteins within the ER lumen. This triggers ER stress. In response to this stress, an unfolded protein response (UPR) is activated as a mechanism to cope with the stress and restore internal balance. The UPR is regulated by three sensors located in the ER: inositol‐requiring enzyme 1 (IRE1), protein kinase RNA‐like endoplasmic reticulum kinase (PERK), and activating transcription factor 6 (ATF6). However, the UPR can promote tumor growth in vivo by affecting tumor angiogenesis, cell migration, cell metabolism, and treatment resistance, and has a huge impact on the tumor microenvironment.

**Materials and Methods:**

We conducted a literature review of scientific papers on the topic of ER stress in the tumor microenvironment.

**Results and Discussion:**

This review focuses on the inducing factors of ER stress, the mechanism of the UPR signaling pathway induced by ER stress, and the effect of ER stress on the tumor microenvironment and immune‐infiltrating cells. Tumors can regulate their evolution by affecting themselves and the tumor microenvironment through endoplasmic reticulum stress. This study reveals the important role of endoplasmic reticulum stress in the occurrence and development of tumors, and provides new ideas and potential therapeutic targets for the precision treatment of tumors. Future studies can further explore the molecular mechanism of ER stress regulating tumor microenvironment and explore its application potential in clinical diagnosis and treatment.

## 1 Introduction

Endoplasmic reticulum (ER) is a crucial organelle in eukaryotic cells, playing a vital role in the synthesis of proteins, lipids, and steroids as well as calcium‐dependent signaling. Within the ER, translated proteins undergo folding and modification processes. Misfolded proteins are eliminated through ubiquitination and degradation via either the 26S proteasome or autophagy pathways. Hypoxia, oxidative stress, accumulation of reactive oxygen species (ROS), inadequate amino acid supply, Ca^2+^ imbalance, and acidosis can disrupt protein folding within ER, leading to an accumulation of misfolded proteins. When this accumulation exceeds their capacity for degradation, ER stress is triggered, which activates intracellular signal transduction pathways called UPR aimed at the dynamic adjustment of ER folding and degradation capacity and restoration of ER homeostasis [1].

## 2 Molecular Pathways Linked to the UPR

**FIGURE 1:**
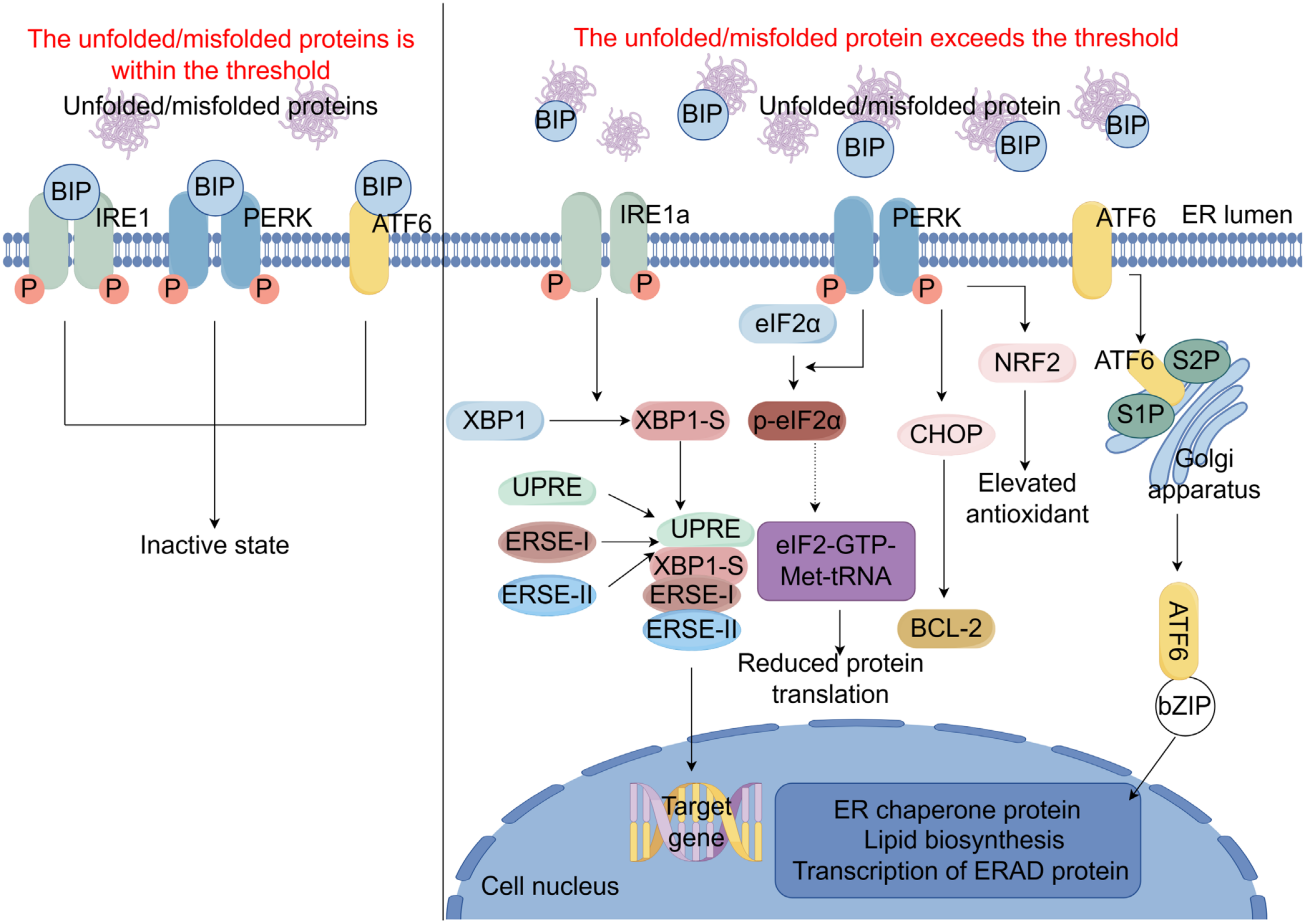
Three branching pathways of ER stress. IRE1α, PERK, and ATF6 bind to GRP78/BIP in an inactive state. When the amount of misfolded proteins in the ER exceeds a threshold, GRP78/BIP is disassociated from the ER stress sensor and IRE1 is phosphorylated to IRE1α. IRE1α subsequently activates XBP1 mRNA, cleaving it into active XBP‐1s. After binding to UPRE and ERSE‐I and ERSE‐II outside the nucleus, XBP‐1s crosses the nuclear membrane and enters the nucleus to induce downstream target genes to exercise their functions. PERK is activated after autophosphorylation upon dissociation of GRP78/BIP, which phosphorylates downstream eIF2α and prevents the binding of the eIF2‐GTP‐Met‐tRNA complex and reduces protein translation. PERK can also phosphorylate NRF2 and transcriptionally upregulate antioxidants. When the above measures still cannot alleviate ER stress, the activation of CHOP downstream of PERK increases the expression of pro‐apoptotic BCL‐2 family proteins and promotes cell death. ATF6 dissociates from GRP78/BIP and translocates to the Golgi apparatus. S1P and S2P localized in the Golgi cleave ATF6 to release the bZIP domain. Translocation of this domain to the nucleus induces ER chaperone proteins, lipid biosynthesis, and transcription of ERAD proteins (by Figdraw).

The UPR is initiated by three types of transmembrane proteins in the ER, which possess the ability to detect misfolded or unfolded proteins within the ER lumen. These proteins include IRE1α, PERK, and ATF6 [2]. Under normal ER homeostasis, IRE1α, PERK, and ATF6 are bound to binding immunoglobulin protein/glucose‐regulated protein 78 (BIP/GRP78) in an inactive state. Due to BIP/GRP78's higher affinity for misfolded proteins, when the quantity of misfolded proteins exceeds a threshold within the ER lumen, BIP/GRP78 dissociates from the ER stress sensors and triggers UPR activation [3]. The UPR restores balance by reducing protein synthesis and increasing the area of the ER to accommodate more folded proteins, which is a homeostatic UPR to protect cell function [4]. However, under severe ER stress conditions where these compensatory mechanisms fail to restore balance, activation of the UPR signaling pathway will induce cell apoptosis [5]. Each type of transmembrane protein that initiates UPR possesses its own distinct molecular mechanism (Figure 1).

### 2.1 The IRE1–XBP1 Signaling Pathway

IRE1 possesses both serine/threonine kinase activity and ribonuclease activity. In mammals, there are two isoforms of IRE1, namely, IRE1α and IRE1β [6]. Because IRE1α is more widely expressed than IRE1β, current mammalian UPR studies favor the IRE1α pathway [7]. Under normal physiological conditions, IRE1α remains inactive by binding to GRP78/BIP [8]. However, during ER stress, it dissociates from GRP78/BIP and undergoes self‐phosphorylation to become activated as IRE1α [8]. Activated IRE1α then initiates the transcription of x box binding protein 1 (XBP‐1) mRNA while cleaving it into an active form known as XBP‐1s. This transcription factor translocates across the nuclear membrane to the nucleus and helps alleviate ER stress by activating processes involved in protein folding, secretion, maturation, and other processes [8].

### 2.2 The PERK Pathway

PERK, a type I transmembrane protein with cytoplasmic kinase activity, exhibits autophosphorylation upon dissociation from GRP78/BIP during ER stress. This autophosphorylation triggers its activation, which then leads to the phosphorylation of eukaryotic translation initiation factor 2α (eIF2α). As a result, the binding of the ternary eIF2–GTP–Met‐tRNA complex is inhibited, leading to a reduction in protein translation and a decrease in the load on the ER [9]. Moreover, PERK can induce nuclear factor erythroid 2‐related factor 2 (NRF2) activation and promote the transcriptional upregulation of antioxidant genes [10]. If the above measures fail to alleviate ER stress, the CCAAT enhancer binding protein homologous protein (CHOP), which is downstream of PERK, is activated and increases the expression of pro‐apoptotic proteins of the BCL‐2 family, thereby promoting cell apoptosis [11].

### 2.3 The ATF6 Signaling Pathway

After separating from GRP78/BIP, ATF6 relocates to the Golgi complex and undergoes cleavage by the Golgi‐specific proteases S1P and S2P. This results in the liberation of its own cytoplasmic basic leucine zipper (bZIP) domain. Upon translocation into the nucleus, the bZIP domain expands ER capacity, boosts ER‐associated degradation pathways, and promotes protein folding efficiency by stimulating ER chaperones, lipid synthesis, and transcription of ERAD proteins [11].

## 3 Relevant Factors Inducing ER Stress in the Tumor Microenvironment

### 3.1 Hypoxia

Hypoxia is a common characteristic of the tumor microenvironment, disrupting the balance within the ER and causing ER stress [12]. Although oxygen is not required for the synthesis of disulfide bonds in proteins, it does play a role in posttranslational folding or isomerization. As a result, hypoxia can lead to abnormal posttranslational folding processes, which in turn induce ER stress [12]. Additionally, oxygen plays an important role in lipid desaturation, and cells experiencing hypoxia have a reduced capacity for desaturation, also limiting ER expansion and triggering ER stress [13].

### 3.2 Disorders in Energy Metabolism and Nutrient Supply

Tumor cells often experience energy dysfunction and ER homeostasis disorders due to their high proliferation and metabolic rates. The dysregulation of intracellular glucose and glutamine plays a significant role in inducing ER stress. Under elevated cellular metabolism, glutamine and glucose deprivation inhibit the hexosamine biosynthetic pathway (HBP), resulting in reduced production of uridine diphosphate‐N‐acetylglucosamine (UDP‐GlcNAc) required for N‐linked glycosylation and ER protein folding [14, 15]. In addition, glucose limitation not only affects ATP and phosphate donors but also inhibits the SERCA pump, thereby activating pathways associated with ER stress [16, 17].

Inadequate supply of amino acids can also trigger ER stress. When amino acids are depleted, activation of eIF2α by GCN2 kinase inhibits protein synthesis and activation of the integrated stress response (ISR) [18]. In addition, excessive consumption of fatty foods rich in long‐chain fatty acids, such as stearic acid, can affect the size, composition, and fluidity of ER membranes. This can disrupt calcium homeostasis and protein glycosylation processes, ultimately causing ER stress [19, 20].

### 3.3 Intracellular ROS Accumulation

Protein folding in the ER depends on its redox state. Under adverse conditions, the concentration of ROS increases, which can disrupt the folding, modification, or degradation of proteins associated with the ER and lead to ER stress [21]. For example, reducing the export of glutathione and modulating the REDOX environment in the lumen of the ER can limit the levels of glutamine and exacerbate ER stress [22]. Additionally, during the process of fatty acid β‐oxidation (FAO), ROS can be generated as by‐products on the inner membrane of mitochondria through the electron transport chain (ETC) [23] or activated downstream by pattern recognition receptors (PRRs) found in pro‐inflammatory cytokines and growth factors to induce NADPH oxidases (NOXs), resulting in excessive ROS production. When intracellular ROS accumulates, it promotes the generation of lipid peroxidation by‐products and regulates calcium channels located within the ER to induce ER stress [24].

### 3.4 Dysregulation of Ca^2+^ in ER

The cellular destiny is regulated by the calcium balance in the ER lumen [25]. Intraluminal Ca^2+^ release from the ER stimulates mitochondrial Ca^2+^ uptake, thereby increasing mitochondrial respiration and ATP production. This process enhances mitochondrial function and promotes cell survival [26]. Subsequently, SOCE replenishes the decreased levels of Ca^2+^ within the ER lumen by facilitating its influx from outside the cell. This mechanism, known as capacitive or storage‐operated calcium entry, is activated through stromal interaction molecule (STIM) [27]. The SERCA pump is a transmembrane protein that utilizes ATP to transport cytosolic Ca^2+^ into the ER lumen [27]. SERCA pumps are encoded by three gene families: SERCA1, SERCA2b, and SERCA3 [28]. Once SOCE completes restoring depleted ER luminal Ca^2+^, calnexin (CNX) and calreticulin (CRT), which are located within the ER lumen, bind to SERCA2b to inhibit its activity for maintaining proper control over Ca^2+^ homeostasis [29]. However, under pathological conditions such as imbalanced uptake/release of ER luminal Ca^2+^, impaired SOCE or SERCA pump function, increased activity of IP3R, and RyR channels responsible for releasing stored intracellular Ca^2+^, as well as dysregulation in Ca^2+^‐binding proteins, can lead to elevated levels of mitochondrial and cytosolic Ca^2+^ while decreasing levels within the ER lumen [28]. Intraluminal reduction of Ca^2+^ disrupts the functioning of chaperone proteins that rely on Ca^2+^ and decreases the threshold for accumulation of unfolded proteins in the ER, resulting in ER stress [30]. Elevated levels of mitochondrial and cytosolic Ca^2+^ can trigger apoptosis by activating the Ca^2+^‐dependent mitochondrial permeability transition pore, as well as enzymes like CaMKII and calpains [31]. In situations involving imbalances in Ca^2+^ and malfunctioning mitochondria, excessive intraluminal Ca^2+^ is transferred from the ER to mitochondria through IP3R located on the ER membrane and calcium uniporter complexes situated on the inner mitochondrial membrane [32]. Elevated mitochondrial calcium can increase ROS and open the mitochondrial permeability transition pore, causing swelling of mitochondria, disruption of membrane potential, rupture of the outer membrane, and release of the proapoptotic factor cytochrome c [33]. The formation of a complex called apoptosome is triggered by the release of cytochrome and the binding of apoptosis activation factor 1; subsequent recruitment induces the activation of initiating caspase, caspase 9, and its downstream effector caspase, triggering apoptosis [34]. Additionally, there exists an interaction between calcium overload and the accumulation of ROS [35]. ROS can enhance the permeability of ER membranes for releasing stored calcium into the cytoplasm, inducing calcium overload [21]. Simultaneously, heightened concentration of cytosolic calcium can be absorbed by mitochondria, which then regulate tricarboxylic acid cycle and ETC enzymes for generating superoxide O2‐, rapidly converting it into ROS, thereby leading to a detrimental cycle [36].

### 3.5 The Accumulation of Lactic Acid

Cancer cells often heavily depend on anaerobic glycolysis as their primary metabolic pathway to maintain a high rate of metabolism, resulting in the production of lactate and subsequent acidification of the tumor microenvironment (TME). When exposed to acidic conditions, specific receptors can activate three signaling pathways associated with UPR in cells [37, 38]. The exact mechanism behind this phenomenon remains unclear but could potentially involve disturbances in intracellular calcium balance or excessive generation of ROS [38, 39, 40, 41].

**FIGURE 2:**
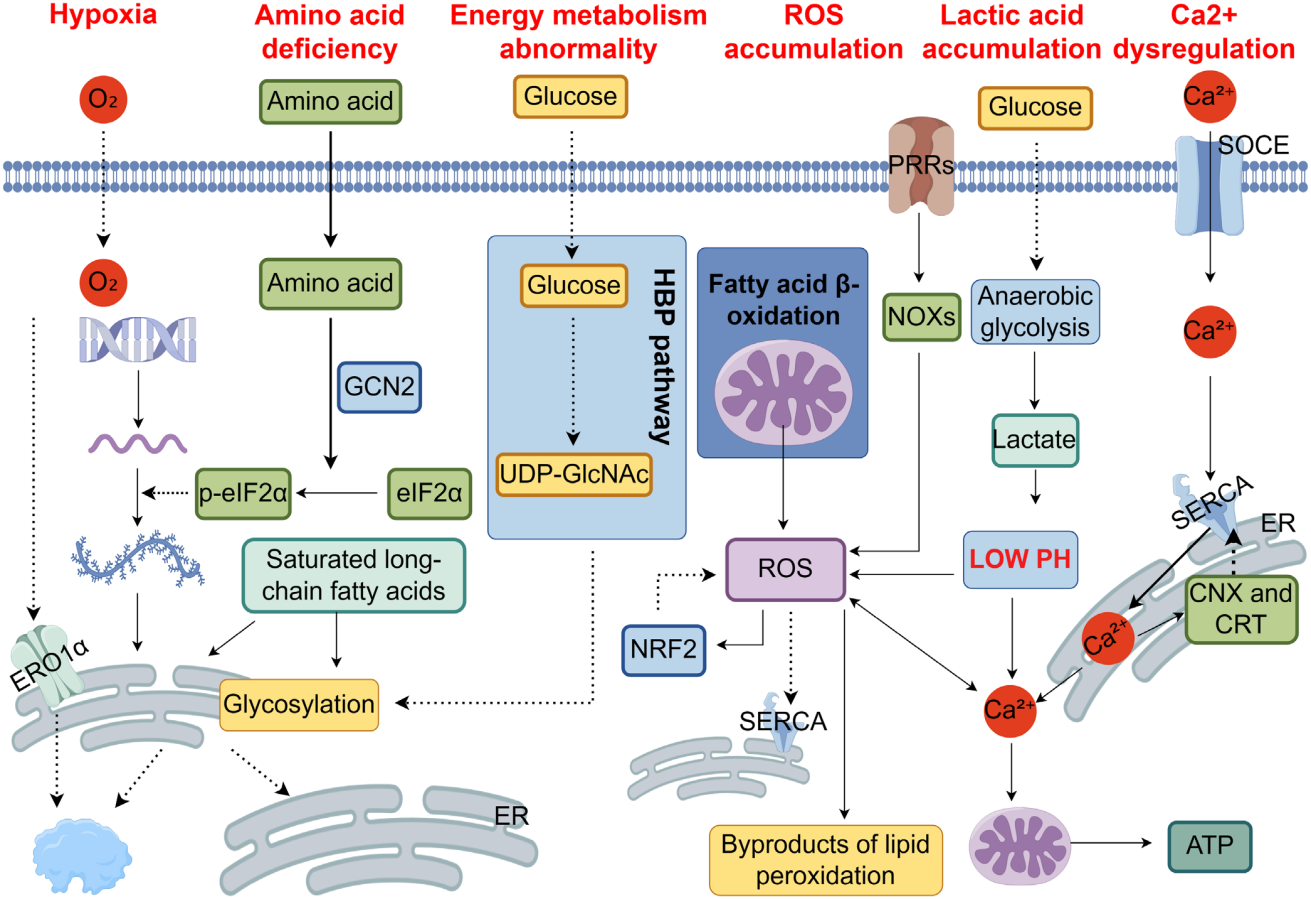
Factors inducing endoplasmic reticulum (ER) stress. The triggering factors of ER stress include hypoxia, disturbances in energy metabolism and nutrient function, accumulation of ROS, Ca^2+^ imbalance, and lactate accumulation. Hypoxia leads to abnormal folding of proteins and also affects the functionality of ERO1α, as well as impairs ER desaturation function, resulting in restricted ER expansion. Insufficient amino acid supply causes phosphorylation of elF2α by GCN2 kinase, thereby reducing protein translation. Inadequate energy supply results in a decrease in UDP‐GlcNAc production through the HBP, limiting protein folding and glycosylation. Saturated long‐chain fatty acids also affect ER fluidity and glycosylation. During fatty acid β‐oxidation, PRRs induce downstream NOXs as well as other undesirable factors; excessive ROS accumulates, inhibiting the resident calcium channel on the ER membrane and leading to ER stress; and accumulated ROS induces activation of NRF2 to limit oxidative damage caused by ROS. SOCE, SERCA pumps, CNX, and CRT are all involved in regulating ER calcium homeostasis. Accumulation of ROS can interact with Ca^2+^ overload; excessive Ca^2+^ within mitochondria increases ROS production, while accumulated ROS can increase mitochondrial permeability, inducing Ca^2+^ overload. Cells generate lactate through the anaerobic glycolysis pathway to lower pH levels in their environment, which disrupts intracellular Ca^2+^ homeostasis or induces excessive ROS production, causing ER stress (by Figdraw).

Relevant factors inducing ER stress in the tumor microenvironment are summarized in Figure 2.

## 4 The Role of Molecules Related to ER Stress in Cancer

### 4.1 BIP/GRP78

Several studies have shown that BIP is overexpressed in different types of human cancers, which contributes to tumor growth through multiple mechanisms. These mechanisms include the enhancement of growth factor maturation and secretion, inhibition of apoptosis, and promotion of angiogenesis [42]. Notably, a portion of the BIP domain can be exposed on the surface of cancer cells; by binding to other receptors, it can mediate downstream signaling cascades [43], for example, the expression of cell surface GRP78 in a variety of cancers, such as leukemia and pancreatic cancer [44, 45]. Furthermore, it binds to the multifunctional cell surface protein Cripto, thereby promoting prostate cancer progression via the inhibition of transforming growth factor‐β (TGFβ) signaling [46].

### 4.2 IRE1α

IRE1 signaling is relatively conserved within UPR pathways, and IRE1α–XBP1s are involved in several cancer cell processes, including proliferation, epithelial–mesenchymal transition (EMT), migration, and invasion [47]. For example, in colorectal cancer, IRE1α‐XBP1s significantly promotes tumor cell invasion and migration ability by regulating key gene expression [48]. Research studies have provided evidence that ER stress induced by hypoxia in colon cancer cells results in a downregulation of WNT/β‐catenin signaling; this downregulation leads to the transcription of hypoxia response genes mediated by XBP1s, which facilitate tumor survival [49]. Additionally, the IRE1α–XBP1s pathway is involved in maintaining ovarian cancer stem cells (CSCs). In human ovarian CSCs specifically, the transcription factor FOXK2 binds to ERN1 elements causing an upregulation of IRE1α expression. This upregulation subsequently induces stem cell–related gene expression mediated by XBP1s and promotes tumor invasion and metastasis [50].

The function of RIDD in the progression of tumors lies in its ability to hinder the splicing of XBP1 mRNA and initiate specific cleavage by IRE1α when there is severe ER stress, thereby breaking down mRNA [51] that encodes proteins related to the ER and limiting the amount of protein within the ER lumen to support cell survival [52]. Furthermore, RIDD also has an impact on reducing angiogenesis and the migration of cancer cells [53]. In summary, cells with high signaling from XBP1s and low signaling from RIDD have lower rates of survival, indicating that the balance between these two pathways determines the fate of cells [54]. In addition, IRE1α can interact with TNF receptor‐associated factor 2 (TRAF2), resulting in the activation of the JUN N‐terminal kinase (JNK) pathway by inhibiting the activity of BCL‐2 and inducing the function of BIM, which ultimately promotes apoptosis [55].

### 4.3 PERK

PERK modulates the progression of tumors through phosphorylation of NRF2, resulting in its separation from the KEAP1 complex. This subsequently triggers the activation of defensive antioxidant responses and ultimately boosts the expression of glutathione, an antioxidant that effectively manages oxidative stress [56]. Furthermore, PERK can promote tumor growth by activating ERO1α and increasing protein folding capacity in the ER [57, 58].

### 4.4 ATF6

ATF6 is a key regulator of tumor growth, malignant progression, and chemoresistance; additionally, it has an impact on autophagy and the microbiota [59, 60]. Studies have revealed that ATF6 increases the expression of protein phosphatase 2A (CIP2A) in colon cancer cells, thereby contributing to an unfavorable prognosis [61]. Moreover, investigations conducted on cervical cancer cells have discovered that ATF6 facilitates cellular migration, EMT, and in vitro cell viability [60].

## 5 The Mechanism of the UPR and the Role of UPR in Cancer

When cancer cells experience ER stress due to a variety of adverse factors, a compensatory UPR is initiated, which affects tumor cells by impacting angiogenesis, cell migration, cell metabolism, and treatment resistance.

### 5.1 Tumor Angiogenesis

The limited availability of oxygen, glucose, and other essential substances may limit tumor cell growth and develop ER stress, especially in cells located in the central region of the tumor. To cope with these adverse conditions and restore ER homeostasis, cells activate the UPR, which regulates the transcription and translation of various factors that promote angiogenesis. By increasing vascular endothelial growth factor‐A (VEGF‐A) levels, the three branches of the UPR can promote angiogenesis, enabling rapidly proliferating tumor cells to evade hypoxia [62]. For example, the activating transcription factor 4 (ATF4) interacts with the promoter of VEGF‐A [63], XBP1 increases the expression of VEGF‐A and interleukin‐6 (IL‐6) by enhancing the transcriptional activity of hypoxia‐inducible factor‐1α (HIF1α) [64], and ATF6 stimulates epidermal growth factor (EGF) to facilitate angiogenesis [65]. The BIP/GRP78 component also contributes to enhanced tumor angiogenesis [66]. Furthermore, another ER chaperone known as oxygen regulatory protein 150 (ORP150), regulated by UPR, promotes the secretion of VEGF [67].

### 5.2 Tumor Cell Migration

The UPR could potentially function as a regulatory mechanism governing the migratory behavior of cancer cells in preclinical models. IRE1α enhances the migration ability of multiple myeloma mesenchymal stem cells by promoting filamin A phosphorylation [68]. Additionally, when the ribonuclease activity of IRE1α is inhibited, it leads to an increase in the secretion of proteins rich in acidity and cysteine, thereby facilitating the invasion and metastasis of tumor cells [69].

### 5.3 Tumor Cell Metabolism

High metabolic rate of tumors exposes tumor cells to insufficient energy supply, resulting in the initiation of ER stress and alteration in their metabolic processes [70]. IRE1α activates the UPR. This leads to transcriptional upregulation of XBP1s, which subsequently enhances the HBP responsible for converting glucose into UDP‐GlcNAc that facilitates protein expression [71, 72]. Additionally, XBP1s also stimulate glycolysis by increasing the expression of glucose transporter (GLUT) through HIF1α activation [73].

### 5.4 Treatment Resistance

Although cytotoxic chemotherapy and targeted therapy have achieved good results as the current mainstream anticancer methods, there is still the problem of drug resistance that cannot be overcome. The main reason is that there are many mechanisms of drug resistance, which are mainly divided into the following aspects, namely, inactivation of drugs by activating detoxification pathways, reducing drug carrier activity, improving efflux pump function, increasing pro‐survival protein expression, and enhancing DNA repair mechanisms [74]. There are many studies showing that UPR is critical for drug resistance in cancer. For example, it has been shown that activation of the UPR was linked to resistance to endocrine therapy, molecular targeted therapy, and conventional chemotherapy in breast cancer [75]. In a previous study on colon cancer cells, 5‐fluorouracil (5‐FU) was found to promote colon cancer cell survival through stimulation of the PERK branch of the UPR signaling pathway and chemoresistance of colon cancer cells [76]. Furthermore, the noncanonical PERK–Nrf2 pathway may also activate Nrf2 and make dedifferentiated cells more resistant to treatment through lower ROS levels and drug efflux enhancement [77]. Some studies have found that cells can become resistant to methotrexate and pemetrexed by inducing the production of UPR through multiomics analysis [78]. Moreover, GRP78 was also associated with sorafenib resistance in advanced HCC patients [79]. Up to now, the mechanism of UPR leading to drug resistance is still unclear. Therefore, the pathways by which the UPR contributes to drug tolerance and the contribution of inhibitors targeting these pathways to the efficacy of cancer treatment are still needed.

### 5.5 The Effect of UPR on Tumors

As mentioned above, UPR in cancer cells can affect angiogenesis, and it has important effects on the migration, metabolic activity, and resistance to treatment of cancer cells. However, numerous studies have pointed out that UPR in vivo can support tumor growth by affecting angiogenesis, metabolism, metastasis, and chemotherapy resistance. For example, IRE1α was found to promote breast cancer growth by degrading tumor suppressor microRNAs [80]. Treatment of IRE1α with compound 4μ8C, which inhibits its endonuclease activity, can effectively inhibit breast cancer from spreading and metastasizing [80]. In another study, it was shown that inactivation of p53 and miR‐34a in tumors easily leads to IRE1α–XBP1S–mediated tumor migration and chemotherapy resistance [81]. In addition, in another study, it has been demonstrated that the PERK–ATF4 pathway may enhance the chemoresistance properties of colon cancer cells, which in a mouse model was found to inhibit the growth of colorectal cancer cells when PERK inhibitors were combined with 5‐FU [76]. However, studies on ATF6 found that ATF6 promotes the proliferation and migration through ER stress and MAPK pathways in cervical cancer cells in vitro [60]. Moreover, the lncRNA‐encoded micropeptide XBP1SBM can also promote angiogenesis and tumor metastasis of triple‐negative breast cancer (TNBC) through the XBP1s pathway [82]. In addition, RCN1 was found to be an ER‐resident protein that transduces the c‐MYC pathway by way of the IRE1α–XBP1s signaling pathway and induces resistance to sorafenib and malignant tumor growth in hepatocellular carcinoma [83]. For tumor cells, whether glucose is sufficient or not, cancer cells tend toward the glycolytic route to create lactate for transport and energy, creating a locally sour microenvironment. This microenvironment can hinder the elimination of bladder cancer through immune cells, as well as encourage tumor growth and spread through stimulating the formation of new blood vessels [84]. Together, these studies are evidence that the UPR enhances cancer progression in the body through angiogenesis, metabolism, migration, and treatment resistance.

## 6 Effect of ER Stress on Tumor Microenvironment

Tumor microenvironment refers to the local environment where tumor cells grow and survive, which includes tumor cells themselves, extracellular matrix (ECM), stromal cells (such as fibroblasts, mesenchymal stromal cells, and adipocytes), and immune cells (including T and B lymphocytes, natural killer cells, macrophages, dendritic cells [DCs]) [85]. The tumor microenvironment, which links different cell types together mainly through endocytosis, has a major impact on tumor proliferation, invasion, metastasis, and resistance to therapy. Endocytosis is a process in cell biology that refers to the mechanism by which cells form vesicles through the invagination of their cell membranes to encase and transport extracellular substances, such as molecules, particles, or fluids, to the interior of cells. Many cells in the tumor microenvironment have endocytosis, which are called endocytic cells, including macrophages, DCs, neutrophils, and natural killer cells [86].

**FIGURE 3:**
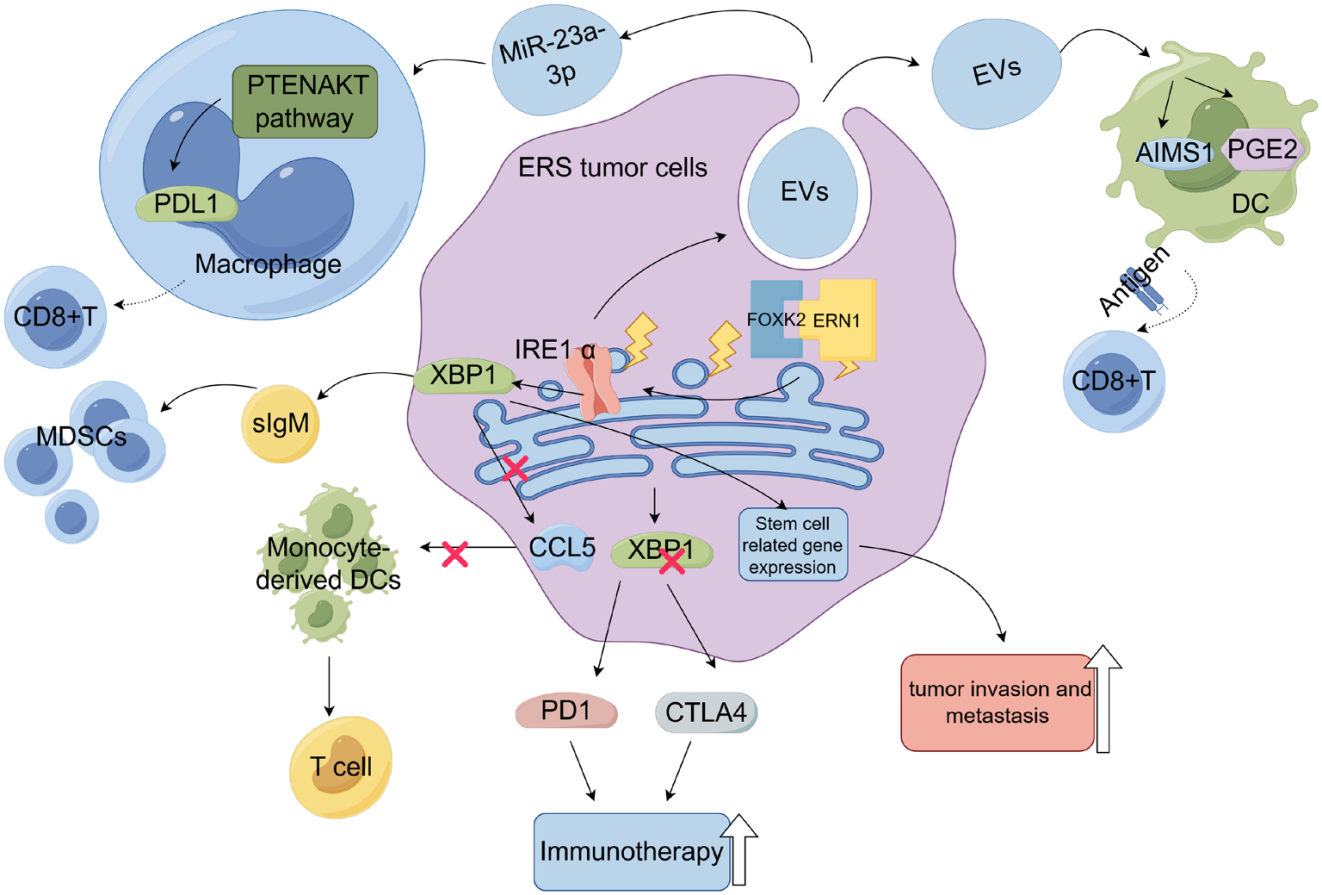
Effect of endoplasmic reticulum stress in tumor cells on tumor microenvironment. Cancer cells release EVs containing special factors under endoplasmic reticulum stress, which upregulate the expression of AIMS1 and PGE2 in DCs and inhibit the ability of antigen presentation to CD8+ T cells. Binding of the transcription factor FOXK2 to ERN1 elements leads to the upregulation of IRE1α expression, which subsequently induces XBP1s to increase the expression of stem cell‐related genes and promote tumor invasion and metastasis. Tumor cells release a large amount of exosomes of miR‐23a‐3p, which upregulates the expression of PDL1 in TAMs and inhibits CD8+ T cells by regulating the PTEN/AKT pathway. IRE1α promotes the generation of EVs and the recruitment of TAMs. Tumor cells promote the overexpression of sIgM through IRE1α–XBP1, thereby promoting the accumulation of MDSCs. In addition, reduced activation of IRE1α–XBP1 inhibits CCL5‐mediated DC recruitment and activation and promotes T cell function. Elimination of XBP1 enhances the immunotherapeutic effect of antibody therapy that blocks PD1 and CTLA4 (by Figdraw).

Tumor cells experiencing ER stress can secrete specific substances that can be taken up by endocytotic cells in the tumor microenvironment, triggering ER stress in endocytotic cells and affecting their ability to infiltrate, a phenomenon known as “infectious ER stress” [87]. Research has shown that these secreted substances from experiencing ER stress mouse models of prostate cancer, lung cancer, and melanoma can promote tumor growth and inhibit immunity by inducing ER stress in myeloid cells within the TME [87]. For example, infectious ER stress increases immunosuppressive arginase 1 (AIMS1) and prostaglandin E2 (PGE2) expression on DCs, thereby reducing the ability of DCs to deliver antigens to CD8+ T cells [88]. In a study on HCC, it was found that treating HCC cells with tunicamycin—an activator of ER stress—could lead to exosomes expressing large amounts of miR‐23a‐3p, regulating macrophage programs via the PTEN/AKT pathway. This regulation affects the production of programmed death ligand 1 (PD‐L1) and subsequently alters macrophage function [89]. In addition, activating ER stress with the ER stress activator thapsigargin was observed to significantly enhance the recruitment and suppressive activity of myeloid‐derived suppressor cells (MDSCs) in a mouse model of colon cancer derived from CT26 cells. The expansion of MDSCs and tumor growth induced by thapsigargin can be mitigated by hormone inhibitors [90]. In addition, ER stress in tumor cells can also affect related functions in infiltrating T cells. Research has found that increased levels of XBP1s within pancreatic tumor cells, together with reduced levels on T cells, synergistically promote the development and spread on metastatic cancer [91]. Analysis of a mouse model lacking ubiquitin ligase 5 (RNF5) revealed reduced UPR gene marker activity in bowel epithelial cells, which may inhibit dendritic cell recruitment and activation mediated by the chemokine ligand CCL5, as well as reduce the formation of antimicrobial peptides [92]; these actions alter gut microbiota composition and promote T cells to reduce the growth of melanoma in mouse models [92]. Additionally, directly suppressing XBP1 in melanoma cells enhances the effectiveness of immunotherapy when treated with antibodies targeting proteins involved in the programmed cell death 1 (PD‐1) and cytotoxic T cell lymphocyte‐associated antigen 4 (CTLA‐4) pathways [92]. Taken together, these investigations demonstrate that tumor cell ER stress can disrupt various mechanisms involved in the immunological reaction in TME, ultimately promoting malignant tumor progression (Figure 3).

## 7 ER Stress Response in Intratumoral Immune Cells

**FIGURE 4:**
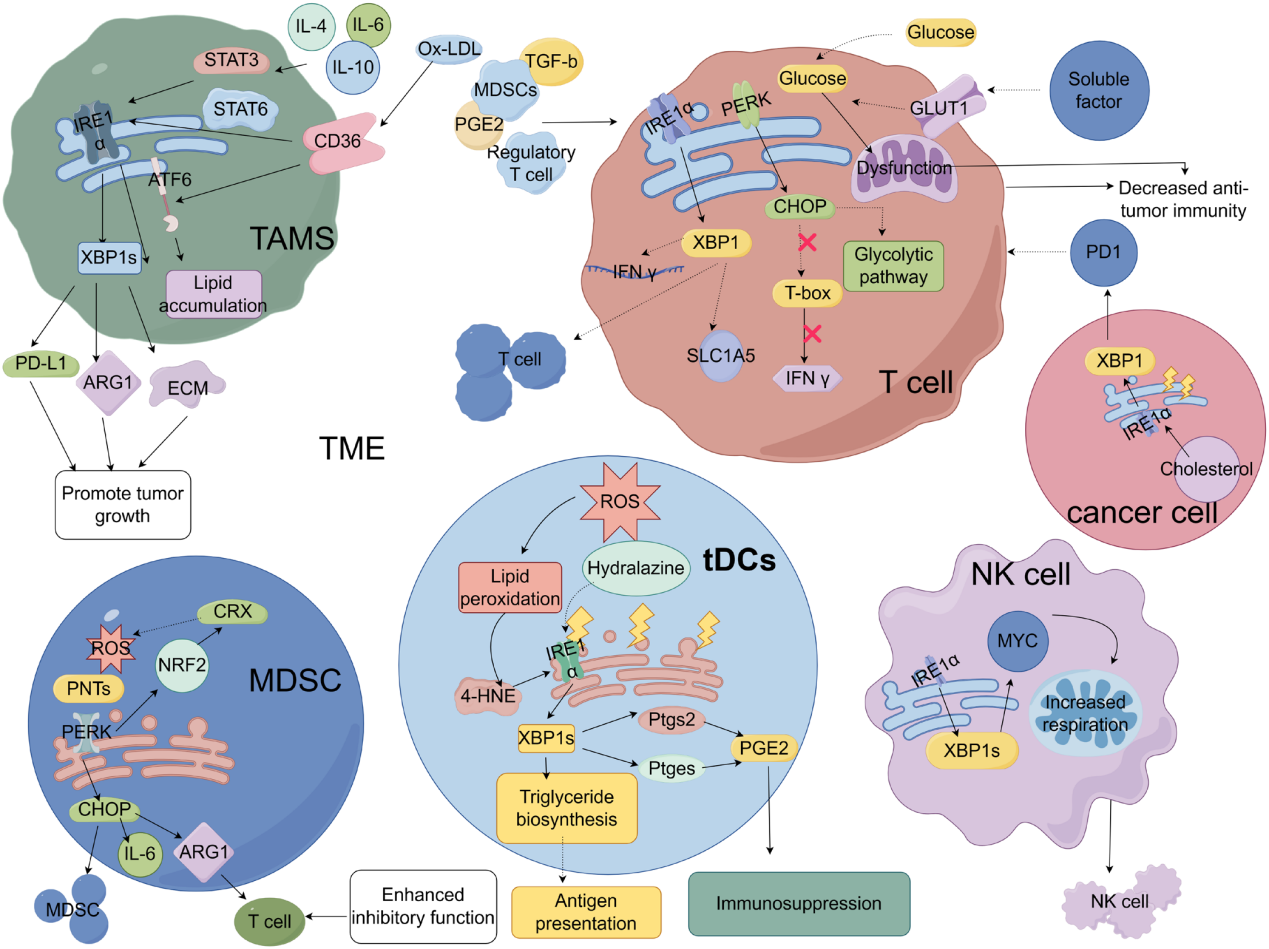
UPR in tumor‐associated macrophages, dendritic cells, myeloid‐derived suppressor cells, T cells, and NK cells. Cytokines IL‐4, IL‐6, and IL‐10, and CD36 in TME trigger IRE1α or ATF6 in TAMs by activating STAT3, STAT6, and uptake of ox‐LDL, respectively, which in turn promote the expression of PD‐L1, ARG1, and ECM and intracellular lipid accumulation, thereby promoting tumor growth. Invasion and immunosuppression. tDCs with accumulation of ROS promote lipid peroxidation and production of 4‐HNE and activates the IRE1α–XBP1 pathway, which transcriptively induces Ptgs2 and ptgges activation and induces triglyceride synthesis, respectively, promoting tumor growth. Hydralazine attenuated the induction of IRE1α–XBP1 by tDCs. ROS accumulation and PNTs activate the PERK–CHOP pathway in MDSCs and increase IL‐6 and ARG1 expression, allowing MDSC aggregation and inhibiting T cell function. PERK can induce CRX to reduce ROS accumulation through NRF2. T cells in the TME are affected by soluble factors, TGF‐b, IL‐10, PGE2, regulatory T cells, MDSCs, and glucose deficiency, which cause ER stress in T cells. XBP1 activation leads to T cell infiltration, IFNG mRNA expression, and reduced SLC1A5 amount. Elevated cholesterol levels in tumor cells activate XBP1, mediate PD1 upregulation, and accelerate T cell exhaustion. ER stress in T cells activates the PERK–CHOP pathway, and CHOP inhibits T‐box, which in turn inhibits IFNγ and reduces glycolysis and mitochondrial respiration in T cells. In NK cells, IRE1α–XBP1 induces MYC and promotes mitochondrial respiration to support NK cell proliferation (by Figdraw).

Owing to the rapid growth and metabolic characteristics of cancer, cancer cells often consume significant amounts of nutrients and energy within the TME, leading to a decrease in TME pH through the anaerobic glycolysis pathway [93]. Under such adverse conditions, it easily inhibits the ability of ER proteins to fold within immune‐infiltrating cells, leading to ER stress and preventing them from exerting effective anticancer effects [94] (Figure 4).

### 7.1 Macrophages Associated With Tumors (TAMs)

Macrophages are part of the innate immune system. They play a key function in regulating immune responses. They compete with dendritic cells for the presentation of antigens and T cell activation. The M1 macrophage subtype is characterized by killing or classical activation, inducing inflammation and anti‐antigen responses. These macrophages release inflammatory factors and eliminate pathogens and unhealthy cells through phagocytosis, while also presenting antigens to activate T cells. Then again, the M2 macrophage subtype is associated with tissue repair or selective activation, suppression of inflammation, and inhibition of the activation of T cells. Factors present within the cancer microenvironment can influence the functions of these two macrophage subtypes, thereby contributing to cancer progression [95].

Cytokines found within the TME, including IL‐4, IL‐6, and IL‐10, trigger signaling pathways involving STAT3 and STAT6 in TAMs and trigger the IRE1α–XBP1 signaling cascade. Activation of IRE1α–XBP1 has been shown to stimulate the release of cathepsins by macrophages in in vitro studies, thus promoting the invasion of cancer cells; this process can be blocked by inhibitors of IRE1α, reducing the tumor invasion caused by macrophages [96]. The scavenger receptor CD36 was reported to cause ER stress within macrophages by taking up oxidized low‐density lipoprotein (ox‐LDL), thereby enhancing receptor expression as well as promoting intracellular lipid accumulation via IRE1α and ATF6 [97]. Lipid‐loaded macrophages have tumor growth promotion and powerful immunosuppression functions [98]. Additionally, investigations on a mouse model with B16F10 melanoma have revealed an activated state of the IRE1α–XBP1 pathway in TAMs. This activation increases the upregulation of the immune regulatory molecules PD‐L1 and arginase 1 (ARG1), thereby facilitating the survival of tumor cells. Furthermore, high expression of CD274, encoding PD‐L1, was associated with an IRE1 alpha‐dependent gene signature in human melanoma samples [99]. Interestingly, mice bearing B16F10 tumors lacking specific IREα expression within their macrophages exhibited significantly improved survival compared to wild‐type mice [99].

### 7.2 Tumor‐Associated Dendritic Cells (tDCs)

Studies have provided evidence that tDCs in murine models of metastatic ovarian tumors exhibit an accumulation in ROS. ROS promotes oxidation on lipids, resulting in the production of highly active by‐products such as 4‐HNE. These by‐products subsequently modify chaperones responsible for retaining proteins within the ER, leading to ER stress and continued engagement of the UPR [100]. The sustained activation of IRE1α–XBP1 in tDCs not only increases the expression levels of various UPR‐related transcription factors but also induces pathways related to triglyceride synthesis and lipid droplet formation. This eventually impairs their capacity to present antigens [100]. Abnormal lipid accumulation and uncontrolled formation of lipid droplets are recognized as important characteristics exhibited by immunosuppressive dendritic cells both in cancer patients and mouse models [101, 102]. The induction of IRE1α–XBP1 in dendritic cells exposed to soluble factors can be reduced by using antioxidant vitamin E or a hydrazine derivative called hydralazine, which chelates 4‐HNE [100]. Additionally, targeting XBP1 specifically in tDCs through therapeutic silencing using nanoparticles or utilizing mouse models with XBP1‐deficient dendritic cells effectively controls abnormal adipogenesis while enhancing antigen presentation in the TME, thereby promoting T cell–mediated antitumor immune responses and delaying ovarian cancer progression in mouse models [100]. It is important to note that dendritic cells identified in human ovarian cancer specimens showed increased expression of genes associated with ER stress, which was linked to reduced T cell infiltration into the TME [100]. Further studies have shown that the IRE1α–XBP1 pathway is involved not only in the control of antigen presentation in dendritic cells but also in the regulation of key immunosuppressive factors. Studies conducted on murine bone marrow–derived dendritic cells and human monocyte–derived dendritic cells have provided evidence that IRE1α–XBP1 promotes the synthesis of various prostaglandins, including the potent lipid mediator PGE2, when subjected to ER stress or PRR stimulation. When activated, IRE1α–XBP1 activates two genes, Ptgs2 and Ptges, which are responsible for the production of PGE2 [103]. Moreover, deletion of either IRE1α or XBP1 in dendritic cells, macrophages, and neutrophils has been shown to impair PGE2 production under conditions of ER stress or inflammation both in laboratory settings and animal models [103]. Subsequently, it was discovered that deleting IRE1α or XBP1 reduces Ptgs2 expression from TNBC cells [104]. Importantly, studies indicate that PGE2 can orchestrate mechanisms leading to immune suppression in cancer [105]. This finding will greatly improve the feasibility of enhancing PGE2 immunosuppression by IRE1α–XBP1 activation in tumor cells and myeloid cells within them to promote tumor malignant progression.

### 7.3 MDSCs

ER stress is crucial in coordinating the immunomodulatory role of MDSCs in tumors. A number of studies suggest that MDSCs, influenced by adverse factors like ROS accumulation and peroxynitrite (PNTs), induce ER stress and activate the PERK pathway of the UPR. Tumor‐infiltrating MDSCs exhibit higher expression levels of CHOP compared to splenic MDSCs from mouse cancer models; this increased expression facilitates the accumulation of MDSCs and enhances their ability to suppress T cell activity by upregulating IL‐6 and arginase expression [106]. Both systemically CHOP‐deficient transgenic mice and CHOP‐deficient bone marrow–reconstituted wild‐type animals show reduced tumor progression. Additionally, when compared to CHOP‐sufficient MDSCs, those derived from tumor‐bearing mice show decreased T cell suppressive function. In conclusion, CHOP is an important regulator of the immune response through its effect on MDSCs [106]. Another study showed that the targeting of PERK in MDSCs can trigger immune responses against tumors mediated by type I interferons in various mouse models of cancer [107]. The activation of PERK in MDSCs enhances their ability to counteract oxidative stress by activating NRF2, a downstream transcription factor; this transcription factor stimulates the production of cellular redox transcripts, thereby reducing the impact of ROS accumulation [108]. Conversely, when PERK is targeted, NRF2 signaling in MDSCs is disrupted and their mitochondrial homeostasis is disturbed, resulting in the cytosolic buildup of mitochondrial DNA127. As a result, this process initiates the generation of stimulator of interferon genes (STING)–dependent type I interferons, which trigger antitumor immune responses [107]. Furthermore, another investigation uncovered that ER stress also affects MDSCs in tumor‐bearing mice to a certain extent [109]. It was observed that compared to neutrophils and monocytes, due to enhanced autophagy through tumor necrosis factor (TNF)–related apoptosis inducing ligand receptors (TRAIL‐Rs) as well as caspase‐8 activity, MDSCs had higher mortality rates and shorter lifespans. The levels of TRAIL‐Rs expressed by MDSCs were found to be linked to the triggering of ER stress, whereas their shortened lifespan facilitated their expansion within the bone marrow [109]. A recent study reported distinguishing low‐density immunosuppressed polymorphonuclear MDSCs (PMN‐MDSCs) from high‐density neutrophils by examining markers such as alterations in ER stress‐related genes [110]. Additionally, triggering ER stress on primary human neutrophils results in a fast elevation of LOX‐1 expression and converts these cells into suppressive PMN‐MDSCs. This transformation process can be reversed by inhibiting IRE1α RNAse [110].

### 7.4 T Cell

T cells can not only combat malignant cells but also generate enduring antitumor immune responses. However, the tumor microenvironment contains various substances that can impact the antitumor immune function of T cells. These substances include TGF‐β, IL‐10, PGE2, regulatory T cells, and MDSCs [111]. Additionally, rapid tumor growth leads to insufficient glucose levels in the surrounding environment, resulting in the dysfunction of immune‐infiltrating T cells; this further impedes their ability to fight against tumors [112]. Research has shown that human ovarian cancer‐derived T cells isolated from samples exhibit extensive XBP1 splicing and increased expression of ER stress genetic markers. These changes are linked to a decrease in T cell infiltration to the tumor site and a decrease in interferon‐γ (IFNG) mRNA expression [113]. Soluble factors are present in the tumor microenvironment of ovarian cancer that can inhibit the upregulation of GLUT1 in T cells, impairing their capacity to uptake glucose effectively. Glucose‐deprived T cells demonstrate deficiencies in both N‐linked glycosylation and reduced mitochondrial respiration and IFNγ production [113]. When glucose is not present, mitochondrial respiration requires glutamine as an essential amino acid. This activates the IRE1α–XBP1 pathway, which reduces the number of glutamine transporters in T cells, thereby limiting glutamine utilization [113]. However, the lack of IRE1α–XBP1 in T cells deprived of glucose resulted in an increase in their mitochondrial respiration and IFNγ production [113]. Mice with ovarian cancer that lacked either IRE1 alpha or XBP1 specifically in T cells also showed a decrease in malignant progression and an improvement in survival rates through the reprogramming of intratumoral T cells [113]. Another study suggested that elevated cholesterol levels in B16 melanoma trigger ER stress in CD8+ T cells, activating XBP1, which then upregulates PD1; this promotes cancer progression by depleting CD8+ T cells within the TME [114]. However, inhibiting XBP1 expression enhances the antitumor activity of tumor‐infiltrating CD8+ T cells [114]. In summary, under nutrient‐rich conditions, IRE1α–XBP1s can restore cellular homeostasis and enhance T cell function but become detrimental to T cells when nutrients are deficient within the TME [115]. Additionally, research has demonstrated that overactivation of the PERK–CHOP pathway impairs the anti‐immune effects of T cells within the TME. In several murine cancer models, CHOP was shown to prevent IFNγ expression in intratumoral CD8+ T cells through direct inhibition of the type 1 helper cell transcription factor TH1 (T‐box) on T cells [116], decrease glycolytic pathway activity, and reduce mitochondrial respiration in CD8+ T cells [116]. However, targeting the PERK–CHOP pathway can increase the antitumor immunotherapeutic effect of T cells in the TME [116, 117].

### 7.5 Natural Killer Cell

Recent research has highlighted the importance of IRE1α–XBP1 in supporting the expansion of activated NK cells in both mice and humans under normal conditions. It has been observed that XBP1s stimulate MYC expression and enhance mitochondrial respiration, thereby promoting the proliferation of NK cells. When B16F10 melanoma cells were intravenously injected into mouse models lacking either IRE1α or XBP1, there was a noticeable decrease in NK cell infiltration within the tumor microenvironment, an increase in lung nodule formation, and reduced survival rates compared to mice with intact IRE1α and XBP1 genes [118]. Unfortunately, the roles of ATF6 and PERK pathways in tumor‐infiltrating NK cells have not been clearly demonstrated (Table 1).

**TABLE 1:** Effect of ER stress on cells in the tumor microenvironment.

Cell types in the TME	Cancer cell type	Effects on cells in the TME	References
TAMs	Gastric cancer	Promotes immune escape	[119]
	Lung cancer	Acceleration of tumorigenesis	[120]
	Melanoma	Immune dysregulation, tumor‐promoting activity	[99, 121]
	Glioblastoma multiforme	Promoting angiogenesis	[122]
	Colorectal cancer	Inhibition of phagocytosis	[123]
	Hepatocellular carcinoma	Promoting tumor growth	[124]
	Breast cancer	Promoting angiogenesis	[125]
T cell	Ovarian cancer	Immunosuppression	[113, 126]
	High‐grade severe ovarian cancer	Depletion of CD8+ T cells	[127, 128]
	Lung cancer	The antitumor ability was decreased	[114]
	Pancreatic ductal adenocarcinoma	Depletion of CD8+ T cells	[129]
tDCs	Ovarian cancer	Immunosuppression	[100]
	Lung cancer	Prevent immune escape of cancer cells	[130]
MDSC	Lung cancer/melanoma/ovarian cancer	Immunosuppression	[107]
	Triple‐negative breast cancer	Reduced immunosuppression	[131]
	Nasopharyngeal carcinoma	Immunosuppression	[132]
	Thymoma/lung cancer/colon cancer/breast cancer	Reduced immunosuppression	[109]
NK cell	Melanoma/lung cancer	The tumor killing ability was enhanced	[133]
	Melanoma	Reduced cell infiltration promotes tumor growth	[118]

## 8 Conclusions

When an excessive buildup of incorrect or unfolded proteins occurs in the ER within cells, it triggers ER stress and activates the UPR. The UPR initiates adaptive reactions to reduce the protein synthesis burden and enhance the clearance pathway for misfolded proteins, aiming to restore cellular balance. However, if ER stress persists, these corrective measures fail to effectively restore proper protein folding in the ER, and the UPR triggers apoptosis, leading to cell death. Several factors cause ER stress in cancer cells, including low oxygen levels, disruptions in energy metabolism and nutrient availability, ROS generation, imbalanced calcium levels, and increased production of lactic acid. On the basis of tumor models, the ER stress–mediated UPR mechanism is mainly manifested in regulating angiogenesis, cellular invasion, metastasis, metabolism, and drug resistance, as well as influencing tumor progression to a certain extent. Moreover, ER stress caused by tumors may also influence the function of immune‐infiltrating cells in the TME to accelerate tumor progression. In addition, under the influence of various adverse factors in the TME, the ER homeostasis of infiltrating immune cells will be destroyed, and infiltrating immune cells may undergo ER stress, which can impair their antitumor immune function and indirectly contribute to tumor progression.

So far, studies have shown that conventional cancer chemotherapy not only has poor efficacy but also easily interferes with ER homeostasis, triggering adaptive ER stress and thereby increasing tumor progression and drug resistance. In conclusion, tumor progression can be inhibited by, for example, inducing severe ER stress or inhibiting the compensatory homeostatic UPR. According to recent studies, the current approach of targeting ER stress to prevent tumor progression is the combination of UPR inhibitors and conventional therapy. This approach has shown significant efficacy in some preclinical cancer models, but unfortunately, its reliability for the treatment of the disease is not clear, and further studies are needed.

## Author Contributions

**Chaosheng Peng:** conceptualization (equal), formal analysis (equal), methodology (equal), project administration (equal), validation (equal), visualization (equal), writing – original draft (equal). **Juan Wang:** project administration (supporting), supervision (supporting). **Shu Wang:** software (supporting), validation (supporting). **Yan Zhao:** supervision (supporting). **Haoyuan Wang:** data curation (supporting). **Yuhao Wang:** resources (supporting). **Yuxuan Ma:** investigation (supporting). **Jianjun Yang:** funding acquisition (supporting), project administration (supporting), writing – review and editing (supporting).

## Ethics Statement

The authors have nothing to report.

## Conflicts of Interest

The authors declare no conflicts of interest.

## Data Availability

No new data were created or analyzed in this study.
